# Employment in India

**Published:** 1888-03-03

**Authors:** 


### EMPLOYMENT IN INDIA.

A probationer with one year's training, aged 20, and a trained nurse of five years' experience in medical and surgical work, aged 25, desire to obtain employment in India. They wish to know what salary they would receive, and if their passage would be paid; also the nature of the work undertaken by the Countess of Dufferin's Committee and those who work under it, and how it differs from that of the Nursing Sisters established by Lady Nora Roberts.

